# Case of Ovarian Tumour

**Published:** 1839-10-19

**Authors:** L. P. Bush

**Affiliations:** Wilmington, Del.


### CASE OF OVARIAN TUMOUR.

By L. P. Bush, M. D., *of Wilmington, Del.*

E. J., female, unmarried, æt. about twenty-two years. Has been, during the last year, much more robust in health than previously. Her parents are both alive, and enjoy health. Has never menstruated normally. The discharge appeared about the usual time of life, but always continued scanty, and at irregular periods, and about two years before the date of first visit, ceased, and has not reappeared. Dysmenorrhœa did not exist. Her appearance is now becoming more masculine; hair of a dark colour is seen about chin, and her voice has recently become rough and masculine.

Saw her first in December, 1837, when the above note was taken. I took from her about 14 oz. blood, and gave pills of aloes and myrrh, which were taken without effect. Both previously to this time and afterwards, she had taken various popular nostrums for amenorrhœa without any effect.

An examination *per vaginam* was now made. Uterus was found low down; os tincæ, and neck of uterus, rather elongated and pointed; not more tenderness than natural.

I saw her again in December, 1838. Her abdomen was distended to the size of a sixth month pregnancy, and resonant on percussion. In each groin, and extending nearly up to the anterior superior spinous process of the ileum, and forward towards the median line, is a tumour, flat on percussion, very tender on pressure, and excruciatingly painful at intervals. Appetite slight; pulse varies from ninety to one hundred; heat of skin above natural. The size of the abdomen varies, the distention increasing soon after taking food.

The tumours were twice leeched, and afterwards blistered, with some relief to the pain, but no decrease in their size. Ext. conium and opium were administered, and tart. antim. in half grain doses, every two hours, while febrile excitement continued. The pain gradually and almost entirely subsided during the two weeks she was under treatment. She was then directed to take 5 grains iodid. potass. three times daily, until ℥j. was taken.

From this time until May 1st, 1839, I heard nothing definite of her. I afterwards learned that she took nearly all the iodid. potass. ordered. During this period she frequently walked to Wilmington, a distance of three miles, without much inconvenience; she was, however, troubled with flatulence, and the tumours gradually increased. There were also occasional discharges of blood from the vagina.

At this time her condition was—countenance pale, emaciated; abdomen enlarged to size of seventh month pregnancy; a tumour hard, irregularly lobulated to the touch, was discovered rising up to the umbilicus; below this point percussion was flat,—above it, resonant; no fluctuation; appetite moderate; pulse rather weak, and more frequent than natural; tongue pale and clean; she suffers violent pain, referred by her to the tumour and lumbar region, and is much increased on pressure; some difficulty of urinating; bowels opened only by purgatives or enemata; a sanious discharge, small in quantity, has been passing from the vagina for several weeks.

An examination *per vaginam* showed the vagina and os uteri in a natural state; the whole uterus could not be felt, the tumour appearing to be develeped in its substance or cavity.

Dr. R. R. Porter was present in consultation—℞. Morph. Sulph. gr. ss. q. 2 vel 3h. This was found at next visit to have afforded temporary relief, and was continued p. r. n. After a few days, the bowels being much constipated, the morphine was diminished, and Ext. Conii gr. v. substituted. An enema was also administered. During the next two weeks the control over the pain was such that the morphine and cicuta were in a great measure discontinued.

During this period her appetite and strength visibly declined; and about the last of May her left foot and leg began to be edematous, and some fluctuation was apparent above the tumour, in the part before occupied by flatus. From this time until her death, she suffered comparatively little pain, being sometimes obliged to resort to laudanum but once in twenty-four or forty-eight hours.

Considerable difficulty of urinating now came on; also dyspnœa and occasional vomiting. An infusion of parsley root, and nit. potash and juniper berries, was directed, without, however, increasing the quantity of urine, improving the urination, or diminishing the rapidity of accumulation. At this time the percussion of the right side of chest, while recumbent, was flat in posterior third, while above there was normal resonance. In the flat portion no respiration was discernible,—above, it was vesicular. Left chest not examined. During the last two weeks of her life she was attended by Dr. James Couper, who punctured her lower extremities, then much swollen, with much relief to the tension, the respiration, and the rejection of food. He also discovered the existence in the vagina of a tumour about the size of a hickory nut, which did not exist at the former examination. Her death occurred on the 5th of July.

*Autopsy twenty-four hours after death.* Dr. Porter, Mr. Delany, of New Castle, and myself, were present on the invitation of Dr. Couper.

The abdomen was much distended, as also were the inferior extremities. Considerable infiltration of the labia externa existed. A dark and very fœtid mass of fungous substance, about the size of a small walnut, protruded into the vagina. The abdomen being opened, the peritoneal cavity was found to be filled with a lye-coloured fluid. Springing from the basin of the pelvis, rising high on the lumbar vertebræ, and extending far over into the right lumbar region, was discovered a large lobated tumour, consisting of eight or ten lobes. The tumour was strongly attached to the cellular tissue covering the muscles of the loins. The posterior portion of the tumour resembled spleen; the anterior portion consisted of several lobes which were connected with one another, and also to the pubis, by false adhesions. This tumour, after being detached, was found to weigh eight pounds. The inferior portion was composed of matter resembling spleen, both in consistency and colour. Internally it consisted of livid and pale-yellow matter, intimately blended with each other, which gave to the whole a mottled appearance. The inferior half of the tumour measured ten inches transversely, and from superior to inferior portion five inches. On the left lateral portion of superior half of the great tumour were two small ones of a pale straw colour; the consistency of upper one was less than that of lower, which was darker and larger. The upper half of great tumour was of a sarcomatous character; on the right side of which were two large lobes of a grayish brown colour, about the consistency of indurated liver. The posterior lobes of inferior half presented the same appearance as that of the anterior ones. Both fallopian tubes were found on the right side of the tumour adhering to its peritoneal covering. The left one was about eight inches in length, the right about six inches; they were pervious throughout their whole extent, and their fimbriated extremities adhered loosely to the tumour. The left tube, commencing on the right side, ran over the superior part of the tumour, and attached itself, as above described, to the left of the middle line, the greater portion of the mass having been developed on the left side. No median line, or separation, could be found in the mass, the two tumours originally observed having coalesced. The *ovaria* could no where be found. The *uterus* was found nearly *in situ*, and deviated from the natural condition only in its length, which was one-half greater than usual. Several small tumours were found in the vagina, besides the large one above mentioned.

The bladder adhered very firmly and extensively to the anterior inferior portion of tumour.

The stomach presented a natural and healthy appearance, except that portion of the mucous membrane immediately around the pyloric orifice, which was of a livid colour, and had an arborescent arrangement of blood-vessels.

The *exterior* surface of the right lung presented about thirty tumours, from the size of a grain of wheat up to that of a small walnut, which possessed a similar character to that of inferior half of great tumour, viz., hæmatic or spleen-like. No tumours were found in the interior of the lungs. Extensive false adhesions were found on the posterior portion of left lung. This lung presented the same appearance as the right. The right pleura contained about a quart of dark-coloured fluid,—the left not quite so much.

On the posterior portion of right lobe of liver was a tumour of the size of a large walnut, the structure of which was the same as those in the lungs. Developed within the liver were, also, numerous other small tumours of the same nature.

The substance of the kidneys was unusuall pale.

Mesenteric glands were about the natural size, but universally darkened.

Small intestine contained a fluid of a bright yellow colour. The mucous membrane, low down in the jejunum, was considerably softened; elsewhere, normal.

*Remarks.*—It will be perceived from the preceding account, that the treatment adopted produced no perceptible effect on the progress of the disease. By it the unhappy patient was rendered more comfortable, but the malady marched steadily onward to its consummation. The iodine seemed to be attended with no advantage appreciable to the parents, the appetite appearing unaffected in any way; no diminution or even retardation of the growth of the tumour, nor yet was there general emaciation.

We may infer from the fact of the patient having never menstruated normally, that a diseased condition of the ovariæ existed at puberty, although it was not manifested externally until several years afterwards. From the period when the tumour was perceived above the pubis, until death, embracing more than seven months, a much greater portion of the tumour had been formed than during the whole previous period, whatever it may have been since its growth commenced; so that the decline of her health, and the growth of the morbid mass, were coexistent.

The composition of the tumour presented a specimen of scirrhous, encephaloid, and hæmatoid formation, seeming to prove that these structures are very closely allied in their characters, since here in the same mass, nourished by the same vessels, was found each of these structures as perfect as it could have been produced, had the tumour been composed of one only. There was also found a structure strongly resembling in colour and consistence boiled liver. The prevailing degeneration was the hæmatoma or spleen-like. Here we remark a notable difference between this case and that reported by Dr. Pennock in No. 30 of the Examiner. The disease in this case invaded almost all the important organs of the system; in the one referred to, it was only in the glands of the meso-colon that any scirrhous formation was detected. In the liver, the tumours pervaded its structure, but in the lungs they were found only beneath the pleura pulmonalis; and, being scattered over its surface, affected neither the percussion nor respiration in a sensible degree.

Another important distinction between these two cases is found in the entire absence of pain in that by Dr. P., while in this it was endured in an extreme degree.

Several other cases which have come under my notice, presented this difference. In one which occurred in the Philadelphia Hospital, in a young woman, (Turner,) the pain was sometimes excessive, so that on one occasion ʒss. Sulph. Morph. was administered in a single night, and Ext. Cicuta ʒj. was her daily dose. In another case, an aged female, a cicuta plaster sufficed to render her comfortable. As regards the diagnosis, the absence of the menstrual secretion, the tumours in the region of the ovariæ, the hoarse voice, and the hirsute chin, led early to the opinion of extensive disease of the ovaria. In Dr. P.'s case, none of these symptoms could be adduced to favour a diagnosis of ovarian disease. He, however, particularly remarks, that "numerous cases could be cited which entirely disprove the idea, that in all cases of ovarian disease the menstrual function is suppressed, and that the continuance of the catamenia indicates the tumour to be unconnected with the uterus."

